# Biomedical question answering using semantic relations

**DOI:** 10.1186/s12859-014-0365-3

**Published:** 2015-01-16

**Authors:** Dimitar Hristovski, Dejan Dinevski, Andrej Kastrin, Thomas C Rindflesch

**Affiliations:** Institute for Biostatistics and Medical Informatics, Faculty of Medicine, University of Ljubljana, Vrazov trg 2, SI-1104, Ljubljana, Slovenia; Faculty of Medicine, University of Maribor, Slomškov trg 15, SI-2000 Maribor, Slovenia; Faculty of Information Studies, Ulica talcev 3, SI-8000, Novo mesto, Slovenia; Lister Hill National Center for Biomedical Communications, U.S. National Library of Medicine, 8600 Rockville Pike, Bethesda, MD 20894 USA

## Abstract

**Background:**

The proliferation of the scientific literature in the field of biomedicine makes it difficult to keep abreast of current knowledge, even for domain experts. While general Web search engines and specialized information retrieval (IR) systems have made important strides in recent decades, the problem of accurate knowledge extraction from the biomedical literature is far from solved. Classical IR systems usually return a list of documents that have to be read by the user to extract relevant information. This tedious and time-consuming work can be lessened with automatic Question Answering (QA) systems, which aim to provide users with direct and precise answers to their questions. In this work we propose a novel methodology for QA based on semantic relations extracted from the biomedical literature.

**Results:**

We extracted semantic relations with the SemRep natural language processing system from 122,421,765 sentences, which came from 21,014,382 MEDLINE citations (i.e., the complete MEDLINE distribution up to the end of 2012). A total of 58,879,300 semantic relation instances were extracted and organized in a relational database. The QA process is implemented as a search in this database, which is accessed through a Web-based application, called SemBT (available at http://sembt.mf.uni-lj.si). We conducted an extensive evaluation of the proposed methodology in order to estimate the accuracy of extracting a particular semantic relation from a particular sentence. Evaluation was performed by 80 domain experts. In total 7,510 semantic relation instances belonging to 2,675 distinct relations were evaluated 12,083 times. The instances were evaluated as correct 8,228 times (68%).

**Conclusions:**

In this work we propose an innovative methodology for biomedical QA. The system is implemented as a Web-based application that is able to provide precise answers to a wide range of questions. A typical question is answered within a few seconds. The tool has some extensions that make it especially useful for interpretation of DNA microarray results.

**Electronic supplementary material:**

The online version of this article (doi:10.1186/s12859-014-0365-3) contains supplementary material, which is available to authorized users.

## Background

The large size of the life sciences literature makes it difficult even for experts to absorb all the relevant knowledge in their field of interest. Sophisticated technologies are needed, and automatic text mining techniques are increasingly used to help access and exploit online textual resources. The most widely used are information retrieval systems such as PubMed, which searches the MEDLINE biomedical bibliographic database. These systems are very efficient and robust. However, in response to a user’s query they do not provide answers (or facts), but a set of documents (citations) that the user has to read in order to extract the required answers. For example, if a user wants an overview of the Parkinson’s disease literature, a PubMed search will return tens of thousands of documents. If the user is interested in treatments for a disease, with some skill it is possible to specify an effective query, but the result will be a set of documents that have to be read.

Question answering (QA) systems, on the other hand, aim at providing answers (known facts). For the above example about the treatment of a disease, a QA system would provide as answers particular drugs that are used to treat that disease or short text passages that contain the answers. The overall goal of QA systems is to allow users to quickly get precise answers with the least amount of reading required.

Evidence-based medicine [1] is an important paradigm in the medical field which encourages clinicians to use the best evidence from scientific research when making decisions, and it stimulates clinicians to ask questions in order to get the evidence. Research has shown that physicians ask several questions of various types per patient encounter [2,3]; however, they have very limited time for seeking an answer, on average less than two minutes. For many questions they do not even try to search for an answer, and even if they try, often the answer is not found [4]. On the other hand, Hersh et al. [5] have shown that at least 30 minutes are needed on average to find an answer. Therefore, many questions remain unanswered.

Clinical medicine is not the only field that needs efficient access to the literature for answers to questions. Genomic research is another example of such a field. With high-throughput technologies, such as genomic microarrays, it is now possible to measure the expression levels of essentially all genes within an entire genome scale simultaneously in a single experiment and to provide information on gene functions and transcriptional networks [6]. However, the successful interpretation of this information for integration into research underpinning biomedical progress is impossible without comparison to the published literature.

In this paper we present a QA tool, SemBT, that we have developed. It is able to answer a wide range of biomedical questions, not only for clinical medicine, but also for medical research in general, including pharmacogenomics and microarray experiment result interpretation. The tool returns answers in a top-down fashion, first very precise answers in the form of semantic relations, and then, on demand, more detailed answers. The tool is very fast and publicly available.

### Question answering work by others

QA can be open-domain [7,8] or closed-domain [9]. Open-domain QA is concerned with questions about nearly anything and is considered as more difficult than closed-domain QA. In open-domain QA, general ontologies and sources of world knowledge are used, and the answers are extracted from very large amounts of data. Closed-domain QA is sometimes also called restricted-domain QA. Unlike open-domain, closed-domain QA is restricted to a particular area, for example clinical medicine. A general review of the characteristics of closed-domain QA can be found in Molla and Vicedo [10]. Zweigenbaum [11] provides a short biomedical QA review. A recent, much more extensive biomedical QA review, is provided by Athenikos and Han [9]. They further divide biomedical QA into medical QA, dealing mostly with clinical aspects, and biological QA, focusing on molecular biology or genomic types of questions. In our approach, we deal with both medical and biological QA. According to Athenikos and Han, our methods can be generally classified as semantics-based biomedical QA, and we will mention relevant work done by others in this area. Jacquemart and Zweigenbaum [12] investigate the feasibility of semantics-based approaches for the development of a French-language medical QA system. Niu et al. [13] report on their EPoCare (Evidence at the Point of Care) project for answering clinical questions. Demner-Fushman and Lin [14] use a series of knowledge extractors, both knowledge-based and statistical, for clinical question answering according to the principles of evidence-based medicine. Weiming et al. [15] use UMLS [16] semantic relations for clinical QA. They also use SemRep [17], but in a different way than we do. They use SemRep and MetaMap [18] for question processing, then a more traditional information retrieval method for candidate answer selection, and finally they again use MetaMap and SemRep to extract concepts and semantic relations from the candidate answers and match them to the starting question. Biomedical question-answering become the focus of the TREC genomics track in 2006 and 2007 [19], with the introduction of a new task that was concerned with the retrieval of short passages to answer questions, together with the retrieval of the passage location in the source document. Cao et al. [20] describe the AskHERMES online system for answering complex clinical questions. The Linked Open Data (LOD) initiative makes large amounts of data from different domains, including biomedicine, available on the Web and accessible through Semantic Web technologies. Querying this distributed and heterogeneous data set is one of the big challenges in this informatics area. Therefore, there has been an increasing interest in question answering over linked data. For the area of biomedicine, there has recently been a challenge called QALD (question answering over linked data) within CLEF [21].

### Microarray text mining

Several statistical techniques have been used to manipulate features in MEDLINE citations on microarray experiments. Shatkay et al. [22], for example, extract gene function terms from a set of citations related to a kernel document using a document similarity algorithm. Most methods use co-occurring text words [23], often along with either additional information such as MeSH indexing or structured information from related databases such as the Gene Ontology [24,25]. Some systems use a thesaurus to identify concepts in text [26] or compute implicit information on the basis of terms related through co-occurrence with shared, intermediate terms [27].

### SemRep natural language processing system

SemRep [17] is a symbolic natural language processing system for identifying semantic predications in biomedical text. The current focus is on MEDLINE citations. Linguistic processing is based on an underspecified (shallow) parse structure supported by the SPECIALIST Lexicon [28] and the MedPost part-of-speech tagger [29]. Medical domain knowledge is provided by the UMLS [16]. Predications produced by SemRep consist of Metathesaurus concepts as arguments of a Semantic Network relation.

SemRep identifies many semantic predications representing various aspects of biomedicine. The core relations addressed refer to clinical actions (e.g. TREATS, PREVENTS, ADMINISTERED_TO, MANIFESTATION_OF) and organism characteristics (LOCATION_OF, PART_OF, PROCESS_OF). SemRep has recently been enhanced to address pharmacogenomics text [30]. Relations in this semantic area refer to substance interactions and pharmacologic effects (AFFECTS, CO-EXISTS_WITH, DISRUPTS, AUGMENTS, INTERACTS_WITH, INHIBITS, STIMULATES), as well as genetic etiology (ASSOCIATED_WITH, PREDISPOSES, CAUSES). The majority of SemRep’s relations are drawn from the Semantic Network; however, several have been defined to extend the coverage of that ontology in several semantic areas, including ADMINISTERED_TO (clinical actions), CO-EXISTS_WITH (substance interactions), and PREDISPOSES (genetic etiology).

Each semantic relation extracted by SemRep is based on an ontological predication contained in a modified version of the UMLS Semantic Network. The arguments in these predications are UMLS semantic types, such as “Human” or “Anatomical Structure”, which can, for example, appear in the predication “Anatomical Structure PART_OF Human.” All predications extracted from text by SemRep must conform to an ontological predication.

Semantic interpretation is based on the underspecified parse structure, in which simple noun phrases are enhanced with corresponding Metathesaurus concepts by MetaMap [18]. For example, processing of the phrase *Extracorporeal shock wave lithotripsy in the treatment of renal pelvicalyceal stones* produces the structure below.

[[mod(extracorporeal), mod(shock), mod(wave), head(lithotripsy), metaconc(‘Extracorporeal Shockwave Lithotripsy’:[top])], [prep(in), det(the), head(treatment), metaconc(‘Treatment’:[top])], prep(of), mod(renal), head(pelvicalyceal stones), metaconc(‘Kidney Calculi’:[patf])]]

The noun phrase *Extracorporeal shock wave lithotripsy* has been mapped to the concept “Extracorporeal Shockwave Lithotripsy” with semantic type “Therapeutic or Preventive Procedure” (topp). The parse structure enhanced with Metathesaurus concepts underpins the construction of a semantic predication. SemRep first applies “indicator” rules which map syntactic elements (such as verbs and nominalizations) to the predicate of an ontological predication. Argument identification rules (which take into account coordination, relativization, and negation) then find syntactically allowable noun phrases (enhanced with Metathesaurus concepts) to serve as arguments of indicators. The semantic types of the Metathesaurus concepts for the noun phrases must match the semantic types serving as arguments of the indicated ontological predication. In the example above, *treatment* is an indicator for TREATS, which has the corresponding ontological predication “Therapeutic or Preventive Procedure TREATS Pathologic Function.” The concepts corresponding to the noun phrases *Extracorporeal shock wave lithotripsy* and *renal pelvicalyceal stones* can serve as arguments of TREATS because their semantic types (“Therapeutic or Preventive Procedure” (topp) and “Pathologic Function” (patf)) match those in the ontological predication. In the final interpretation, ”Extracorporeal ShockWave Lithotripsy TREATS Kidney Calculi,” the Metathesaurus concepts from the noun phrases are substituted for the semantic types in the ontological predication.

## Methods

According to Hirschman and Gaizauskas [31], the processing involved in QA, in general, consists of the following phases: question processing, document processing and answer processing. A user question is the input to the question processing phase. The question is usually specified in natural language, but it can also be specified with predefined question templates. The question is analyzed and classified. The major goal of this processing step is to determine the type of question and the major concepts involved in the question. The output of this phase is an appropriate query which is used as input to document processing, the second phase. A traditional information retrieval system is normally used to retrieve documents satisfying the query. Then, passages are extracted which serve as answer candidates and as input to the last phase, answer processing; in this step, the candidate answers are compared to the user question and ranked by how well they satisfy the user question. Finally, a normally small set of answers are grouped and shown to the user.

The processing involved in our approach differs from the general approach described above. We start with an extensive preprocessing step during which we first extract semantic relations from MEDLINE with SemRep [17]. For us, the extracted semantic relations are elementary answer components, which are used later to answer actual questions. During preprocessing, the extracted semantic relations are organized in a database enriched with additional information, such as information from the UMLS and certain microarray experiments. Also, during preprocessing, additional index structures are built to allow very fast access to the database. This database is the foundation for the rest of our approach. The next phase, question processing, is realized as a search in the database of extracted semantic relations. Finally, in the answer processing phase, we present the resulting semantic relations as answers in a top-down fashion, first semantic relations with aggregated occurrence frequency, then particular sentences from which the semantic relations are extracted, and finally, on demand, the MEDLINE citations from which the sentences come from.

### Preprocessing

During preprocessing, we first extract semantic relations from MEDLINE with SemRep (e.g., “Levodopa-TREATS-Parkinson Disease” or “alpha-Synuclein-CAUSES-Parkinson Disease”). The semantic types provide broad classification of the UMLS concepts serving as arguments of these relations. For example, “Levodopa” has semantic type “Pharmacologic Substance” (abbreviated as phsu), “Parkinson Disease” has semantic type “Disease or Syndrome” (abbreviated as dsyn) and “alpha-Synuclein” has type “Amino Acid, Peptide or Protein” (abbreviated as aapp). During the question specifying phase, the abbreviations of the semantic types can be used to pose more precise questions and to limit the range of possible answers.

We store the large set of extracted semantic relations in a MySQL database. The database design takes into consideration the peculiarities of the semantic relations, the fact that there can be more than one concept as a subject or object, and that one concept can have more than one semantic type. The data is spread across several relational tables. For the concepts, in addition to the preferred name, we also store the UMLS CUI (Concept Unique Identifier) as well as the Entrez Gene ID (supplied by SemRep) for the concepts that are genes. The concept ID field serves as a link to other relevant information. For each processed MEDLINE citation we store the PMID (PubMed ID), the publication date and some other information. We use the PMID when we want to link to the PubMed record for additional information. We also store information about each sentence processed: the PubMed record from which it was extracted and whether it is from the title or the abstract. The most important part of the database is that containing the semantic relations. For each semantic relation we store the arguments of the relations as well as all the semantic relation instances. We refer to semantic relation instance when a semantic relation is extracted from a particular sentence. For example, the semantic relation “Levodopa-TREATS-Parkinson Disease” is extracted many times from MEDLINE and an example of an instance of that relation is from the sentence “Since the introduction of levodopa to treat Parkinson's disease (PD), several new therapies have been directed at improving symptom control, which can decline after a few years of levodopa therapy.” (PMID 10641989).

At the semantic relation level we also store the total number of semantic relation instances. And at the semantic relation instance level, we store information indicating: from which sentence the instance was extracted, the location in the sentence of the text of the arguments and the relation (this is useful for highlighting purposes), the extraction score of the arguments (tells us how confident we are in identification of the correct argument) and how far the arguments are from the relation indicator word (this is used for filtering and ranking). We also wanted to make our approach useful for the interpretation of the results of microarray experiments. Therefore, it is possible to store in the database information, such as an experiment name, description and Gene Expression Omnibus ID. For each experiment, it is possible to store lists of up-regulated and down-regulated genes, together with appropriate Entrez gene IDs and statistical measures showing by how much and in which direction the genes are differentially expressed. We are aware that semantic relation extraction is not a perfect process and therefore we provide mechanisms for evaluation of extraction accuracy. In regard to evaluation, we store information about the users conducting the evaluation as well as the evaluation outcome. The evaluation is done at the semantic relation instance level; in other words, a user can evaluate the correctness of a semantic relation extracted from a particular sentence.

The database of semantic relations stored in MySQL, with its many tables, is well suited for structured data storage and some analytical processing. However, it is not so well suited for fast searching, which, inevitably in our usage scenarios, involves joining several tables. Consequently, and especially because many of these searches are text searches, we have built separate indexes for text searching with Apache Lucene, an open source tool specialized for information retrieval and text searching. In Lucene, our major indexing unit is a semantic relation with all of its subject and object concepts, including their names and semantic type abbreviations and all the numeric measures at semantic relation level. Our overall approach is to use Lucene indexes first, for fast searching, and get the rest of the data from the MySQL database afterwards.

**Figure 1:**
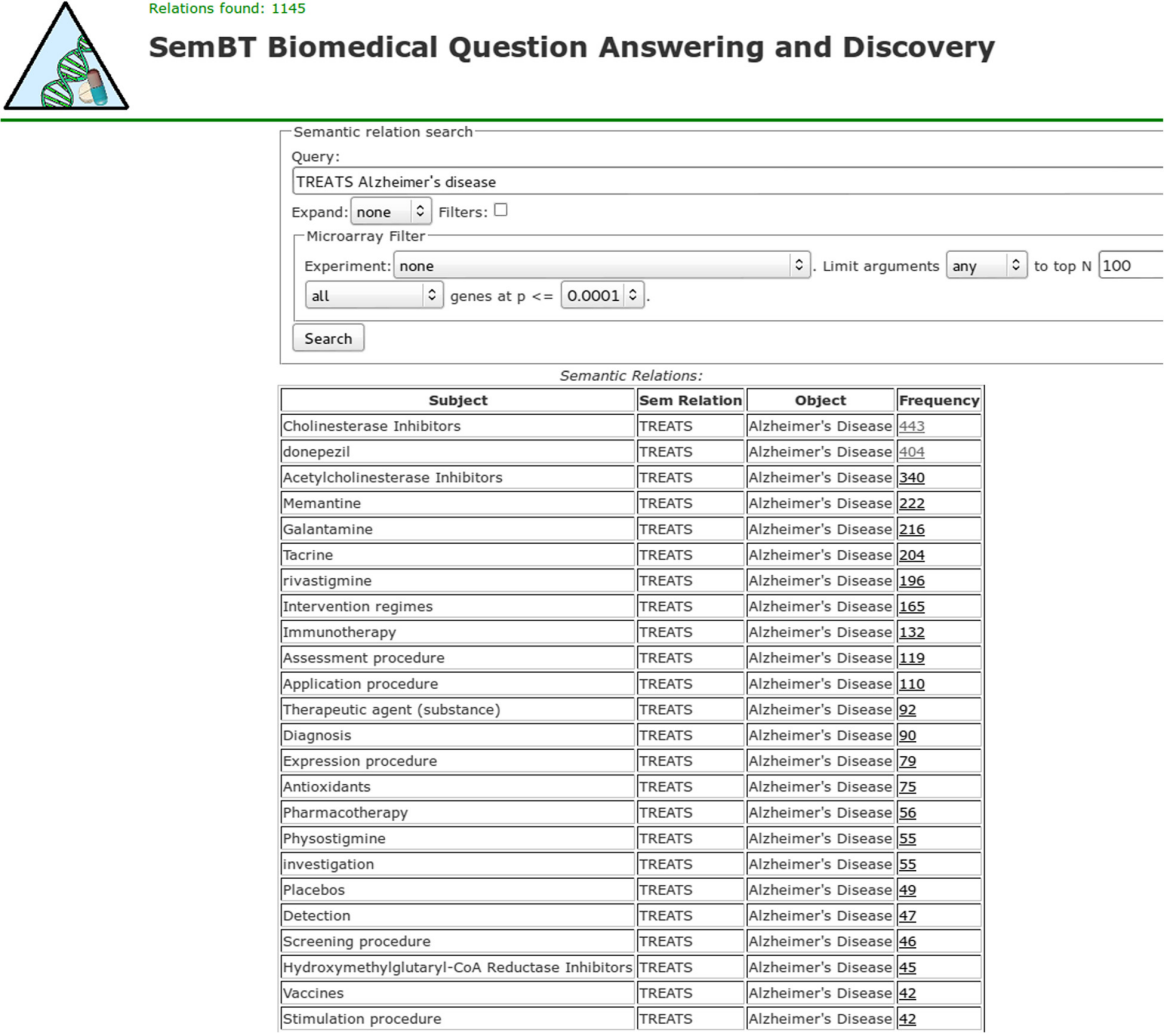
**The user interface of the question answering tool SemBT.**

The QA tool presented in this paper has as a major goal answering what is currently known based on the biomedical literature. The tool is web-based and is one of the several tools available at the SemBT website. The other tools’ major focus is on literature-based discovery, or discovering new knowledge from the literature. The front-end (user interface) for all SemBT tools was developed with the Ruby on Rails application development framework. Figure 1 illustrates the user interface of the QA tool. The details of the types of supported questions and how the answers are presented are described in the next few sections.

### Question processing

In contrast to the usual QA methodology, in this phase we do not create a query for document retrieval, but rather we create a query for searching in the database of extracted semantic relations described in the previous section. In the interface shown in Figure 1, the user enters the question in the “Query” field then presses the “Search” button and gets a list of semantic relations as answers. Right now, the user question (query) cannot be entered in natural language form. In general, the question is specified as a template (subject, relation, object), which refers to various components of the stored semantic relations. At least one component must be specified, but it is possible to specify two or even all three, depending on the question. The question is forwarded to Lucene which means that full Lucene query syntax is allowed. If additional features are used such as query expansion and the microarray filter (described later), the user question is intercepted and reformulated before being submitted to Lucene. In what follows, the most common options when specifying questions are illustrated with examples. A question containing only one of the arguments, e.g. “Alzheimer’s disease” is very general and means any relation between Alzheimer’s disease and some other biomedical concepts. Such question will produce as an answer a set of semantic relations that can be used for a quick overview of a concept. A more realistic question might ask “What are the treatments for Alzheimer’s disease?” and can be specified in our tool in the simplest form as “TREATS Alzheimer’s disease” as shown in Figure 1, in which some of the answers are: “Donepezil-TREATS-Alzheimer’s disease” and “Galantamine-TREATS-Alzheimer’s disease”. The question “What does donepezil treat?” can be asked in our tool as “donepezil TREATS” and the answer will contain, in addition to “Donepezil-TREATS-Alzheimer’s disease”, other relations, including “Donepezil-TREATS-Schizophrenia”. A very specific question such as “Whether donepezil has been used for Down syndrome?” could be asked as “donepezil TREATS down syndrome,” in which all three components are specified; the returned relation “Donepezil-TREATS-Down syndrome” confirms that indeed it has been used for this disorder. When specifying a question, the case does not matter – “Donepezil TREATS Down syndrome” is equivalent to “donepezil treats down syndrome”. The semantic type of the subject and/or object can also be used in questions. For example, “Which pharmacologic substances cause which diseases or syndromes?” can be asked as “phsu causes dsyn”. Here “phsu” is the abbreviation of the semantic type “Pharmacological Substance” and “dsyn” is the abbreviation of the semantic type “Disease or Syndrome”. When specifying a question in the current version of our tool, semantic types must be abbreviated; full names are not accepted. Although this looks like a drawback, the use of semantic type abbreviations has some benefits. One is that the questions become short and simple and the other, more important, is that since the abbreviations are unique and do not appear in the names of the subjects or objects of the relations, it avoids the ambiguity possible when part of the semantic type is found in the subject or object. The semantic relation names that can be used in questions are shown in Table 1. A list of the semantic types and their corresponding abbreviations are shown in Table 2.

**Table 1:** **Top 15 semantic relations extracted with SemRep**

**Predicate**	**Relation count**	**Instance count**
PROCESS_OF	739217	12908669
LOCATION_OF	2033678	9560753
PART_OF	1132906	8736983
TREATS	993417	5435929
ISA	306793	3752806
COEXISTS_WITH	1149505	2496628
AFFECTS	1008068	2124063
INTERACTS_WITH	956926	1824826
USES	225686	1391375
ASSOCIATED_WITH	544318	1316494
CAUSES	525082	1164132
ADMINISTERED_TO	140881	1079967
STIMULATES	442904	845725
INHIBITS	424125	749490
AUGMENTS	384689	742944

Only the top 15 relations with highest instances count are shown [for full table see Additional file 1]. For each semantic relation its name, the number of unique relations and the number of instances are shown.

**Table 2:** **UMLS semantic types and their corresponding abbreviations that can be used for posing questions**

**Abbreviation**	**Semantic type**	**Relation count**	**Instance count**
dsyn	Disease or Syndrome	2603234	12591865
aapp	Amino Acid, Peptide, or Protein	4345793	11503829
podg	Patient or Disabled Group	199404	9258258
bpoc	Body Part, Organ, or Organ Component	1392967	8711584
gngm	Gene or Genome	3422406	6946503
topp	Therapeutic or Preventive Procedure	991912	5108457
neop	Neoplastic Process	802419	4650747
cell	Cell	807092	4346530
mamm	Mammal	456140	4230378
phsu	Pharmacologic Substance	1100842	4226225
orch	Organic Chemical	1550322	3485563
bacs	Biologically Active Substance	951043	2922549
fndg	Finding	592051	2906281
patf	Pathologic Function	667378	2828014
popg	Population Group	339502	2419899
aggp	Age Group	179259	2191630
tisu	Tissue	291668	1634166
celc	Cell Component	349980	1606480
humn	Human	94088	1537291
sosy	Sign or Symptom	275794	1400138
diap	Diagnostic Procedure	271705	1385473
orgf	Organism Function	292346	1307166
inpo	Injury or Poisoning	200300	1076107
celf	Cell Function	370243	1004738
mobd	Mental or Behavioral Dysfunction	186453	997544

Also shown is how many times a semantic type appears as an argument in semantic relations and semantic relation instances. Only the most frequent 25 semantic types are shown out of 133.

In the examples shown above we did not refer to the subject, relation and/or object explicitly, but rather implicitly. A query such as “donepezil treats down syndrome” searches all the words in all the fields of the relations. Most of the time, such a query will be satisfactory; however, it is possible to construct more precise queries by referring explicitly to particular search fields. Subject related search fields are: “sub_name” meaning subject name, and “sub_semtype” meaning subject semantic type abbreviation. Object related search fields are: “obj_name” meaning object name, and “obj_semtype” meaning object semantic type abbreviation. If we do not want to distinguish between the subject and the object, we can use: “arg_name” meaning the name of the subject or the object, and “arg_semtype” meaning the semantic type abbreviation of the subject or the object. And finally, there is one semantic relation related field – “relation” meaning the name of the relation. The query above with explicit search fields would look like “sub_name:donepezil relation:treats obj_name:down syndrome”.

Another implicit aspect of the queries shown so far is the logical connection or operator between the question terms. If there is no explicit logical operator present then AND is assumed. For example, the last query above really means and is equivalent to “sub_name:donepezil AND relation:treats AND obj_name:down syndrome”. In all the examples so far, the actual question is the text between the quotation marks, without the quotation marks themselves. In other words, when constructing questions, it is possible to use the standard Boolean operators AND, OR and NOT and group the search terms with parenthesis. In contrast to the search terms themselves, the logical operators must be capitalized to be properly understood by the tool. The question “What are the genes or proteins known to be etiologically related to Alzheimer?” can be specified with explicit Boolean operators as “sub_semtype:(aapp OR gngm) AND relation:(CAUSES OR PREDISPOSES OR ASSOCIATED_WITH) AND obj_name:Alzheimer” where “aapp” stands for “Amino acid, peptide or protein” and “gngm” for “Gene or genome”. An example of the NOT operator might be the question “What has been used to treat Alzheimer that is not a pharmacological substance?” which could be minimally specified as “NOT phsu treats Alzheimer” and in full form as “NOT sub_semtype:phsu AND relation:treats AND obj_name:Alzheimer”. As practical advice, we recommend that users of our tool first try specifying questions without explicit field reference and Boolean operators. In any case, knowing the names of the available semantic relations and the semantic type abbreviations is essential.

Automatic argument expansion is another useful feature of our QA tool. If requested, it expands the question arguments with semantically narrower concepts (hyponyms). For example, if we issue the query “arg_name:antipsychotic treats” we will get only relations where antipsychotic agents appears. However, if we use argument expansion by selecting from the “Expand” set of options before the query is submitted, the semantic relation ISA (meaning “is a”) is used behind the scenes to search for narrower concepts, and the original query is expanded with them. The results will then also contain particular antipsychotic agents, such as clozapine, olanzapine, risperidone, haloperidol and so on. As another example, we can deal with a whole class of disorders in a question such as “What are the most common treatments for neurodegenerative disorders?” This question can be answered by using expansion in the query “treats arg_name:neurodegenerative”. Here, “neurodegenerative” is expanded with the particular neurodegenerative disorders, such as Alzheimer’s disease, Parkinson disease and so on. A similar question might be “What are the most common treatments for various neoplasms?” Here again we require expansion and use the query “treats arg_name:neoplasms”. Currently, there are some limitations in the argument expansion facility: explicit field reference must be used (e.g., arg_name, sub_name or obj_name); if there are many narrower concepts, only the first one hundred are used; and finally, only a single word can be used to specify the concepts to be expanded (that’s why we used “antipsychotic” and “neurodegenerative” above). The last limitation means that when using expansion, the single word entered (e.g. “antipsychotic”) is used to search for all the concepts containing that word (e.g. “antipsychotic agents”, “atypical antipsychotic”, “Antipsychotic Medications”, …), and, finally, all the concepts found are expanded. Therefore, although a single word is entered, it is possible to expand on multiple word concepts. These limitations are due to technical issues faced when parsing and modifying the original query, and we plan to remove them in the future.

**Figure 2:**
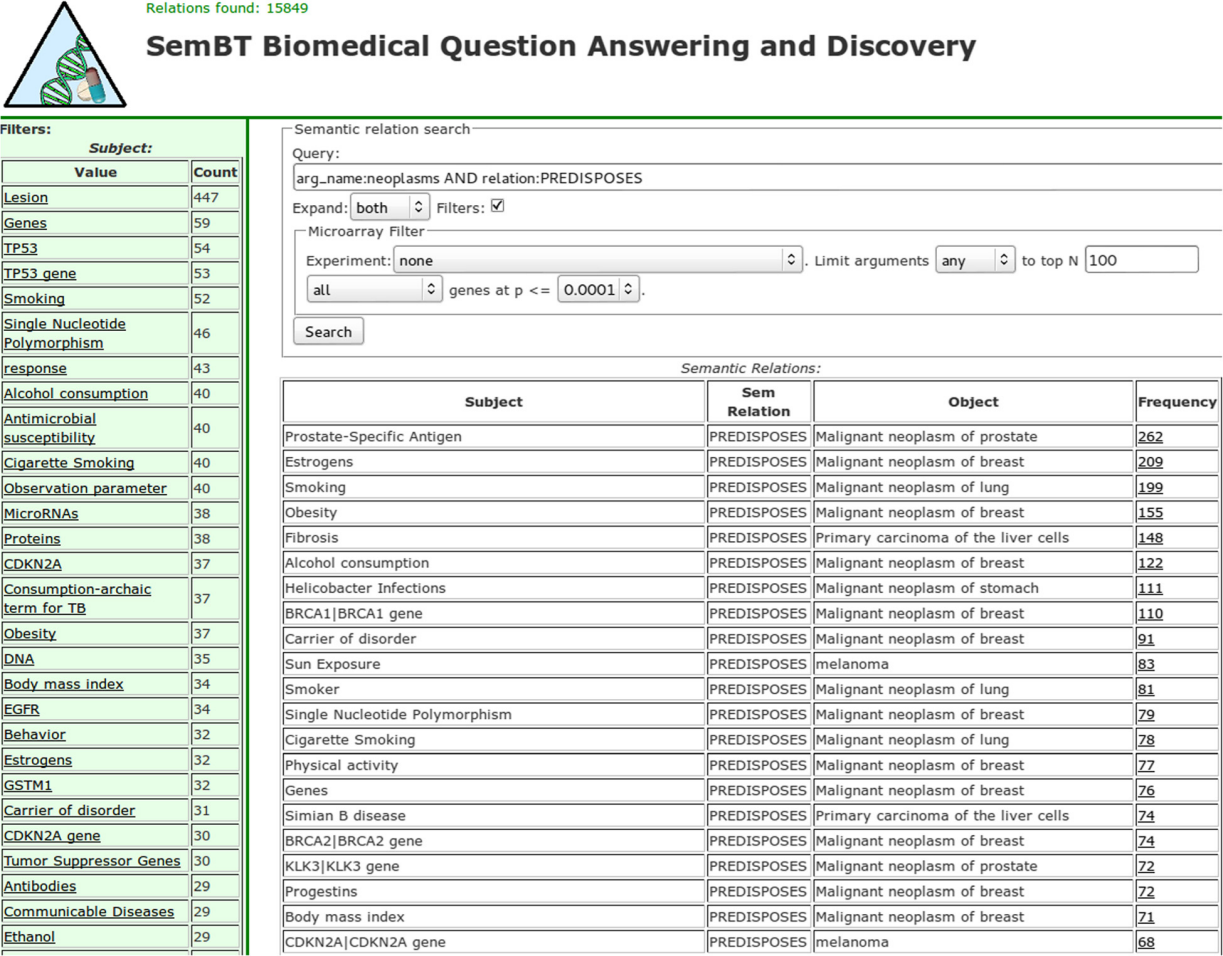
**Faceting, filtering and argument expansion used together to get the factors that predispose various neoplasms.**

When the user question is not specific enough at the beginning or when a more exploratory approach is taken, faceting is another promising avenue to explore. In our tool, faceting is turned on with the “Filter” option and is used for two purposes: to show the top-*N* subjects, relations and objects of a query, and to use these for further query refinement or result filtering. Faceting results are shown in the left column of the user interface (Figure 2). In our faceting approach top-*N* means, in case of the subjects, the top-*N* subjects by the number of relations in which they appear. In other words, a concept that appears as a subject most often in the semantic relations that are the answers to the original query will be shown at the top of the subject facet. The same method applies to the relation and object facets. For example, if the user wants to do some exploratory research on neoplasms and enters the query “arg_name:neoplasms” and also uses argument expansion the most common neoplasms are automatically included in the question. This is a very general question that results in several hundred thousand semantic relations. Now the user can browse the facets in the left column and investigate the subject, relations and objects appearing in highest number of relations. In the relation facet, the PREDISPOSES relation is selected in the relation facet, because that is the aspect the user wants to investigate further. The original query is automatically refined with the selected relation to become “arg_name:neoplasms AND relation:PREDISPOSES” (Figure 2). Now the results of the query show which concepts are known to predispose which particular neoplasms. The facets in the left column can be interpreted as: the concepts in the subject facet are those that predispose the largest number of neoplasms; and the concepts in the object facet are the neoplasms with the largest number of known factors that predispose them.

The QA tool described in this paper is generally applicable in biomedicine; however, it has some extensions that make it especially useful for interpreting microarray results. The problem with microarrays is that although they hold great promise for the advancement of biomedicine, results are not easy to interpret. Microarray experiment results are usually long lists of differentially expressed genes, which can be up- or down-regulated, meaning more or less expressed, respectively, when comparing, for example, a group of patients with a disease with healthy controls. Typically, we want to know as much as possible about the function of these genes. Some of the needed information can be found in specialized genetic databases, but most of it is still in the biomedical literature. In order to make it easier to ask questions about the differentially expressed genes, our tool offers a “Microarray Filter” feature. This filter makes it easy to specify questions about hundreds of genes simultaneously. Here are some example questions: “To which diseases are my up-regulated genes etiologically related?”, “What are the known interactions between the differentially expressed genes?”, “Which substances or other genes can down-regulate my up-regulated genes?” The microarray filter represents a set of conditions to be satisfied in addition to the original user query. The idea of the microarray filter is that options are offered to the user. Based on the selected options, behind the scenes, a few additional queries are issued in order to construct the appropriate complex query. Finally, this query is executed and the results are shown. The first option to select is the microarray experiment. Right now a researcher who wants to use our tool for interpretation has to send us the required data about the microarray experiment (experiment name and lists of up-regulated and down-regulated genes). Then we load these data into SemBT, after which the experiment becomes available in the microarray filter. At this time, there are a few microarray experiments available, but we are working on allowing users to upload their own experiments into the tool directly without our intervention. The next option is selecting which argument of the semantic relations to be limited to the differentially expressed genes. For example, selecting subject here means that only those semantic relations are eligible in which the subject is one of the differentially expressed genes. The next few options allow the selection of up-regulated or down-regulated genes based on several parameters. To illustrate the microarray filter, we describe how the question mentioned earlier (“To which diseases are my up-regulated genes etiologically related?”) can be specified. In the query field we can use “relation: (ASSOCIATED_WITH OR PREDISPOSES OR CAUSES) AND obj_semtype:dsyn”. This query alone would search for semantic relations in which something (not yet specified) is etiologically related (i.e. causes) some disease or syndrome (semantic type “dsyn”). Now we can use the microarray filter to limit the list of semantic relations to only those where the subject is one of the up-regulated genes from one of the experiments. For example, we can first select an experiment (e.g. “Parkinson Meta …”) and then in the field “Limit arguments” we select “Subject” and then “Upregulated”. When the query is submitted, before it is executed, it is modified so that only the up-regulated genes from the selected microarray experiment can appear as subject. The resulting list of semantic relations shows the specific genes and the specific diseases that they cause.

### Answer processing and presentation

**Figure 3:**
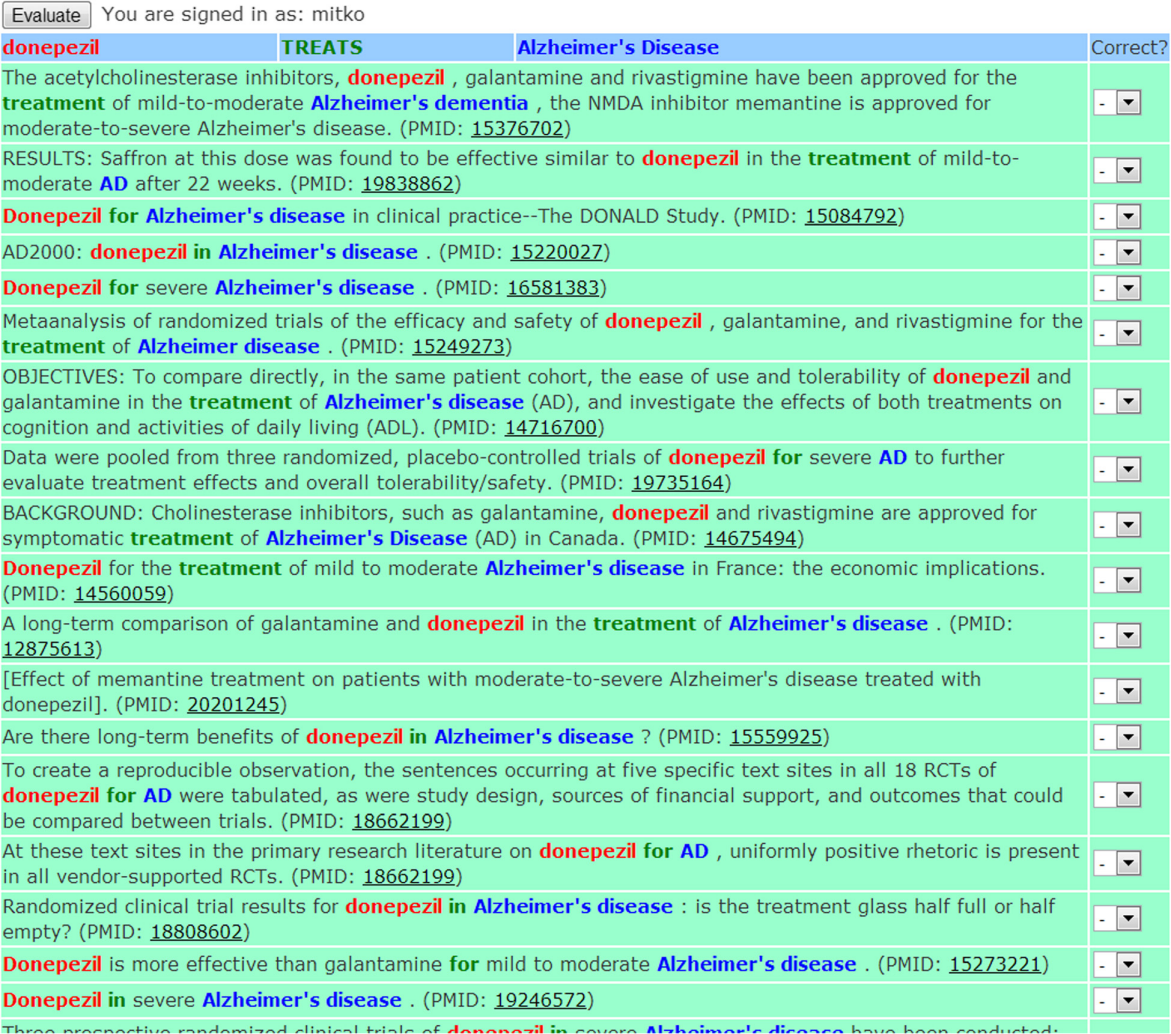
**The first few instances of the semantic relation “Donepezil-TREATS-Alzheimer’s disease” shown as highlighted sentences.**

In the question processing phase the question entered by the user is interpreted depending on user-selected options; then it is executed. Answers are presented in a top-down fashion, semantic relations first, then, on demand, semantic relation instances, and finally, MEDLINE citations. In Figures 2 and 3 in the lower right is the list of semantic relations, which are presented first. In addition to the subject, relation and object fields, the table also contains a “Frequency” field which is the number of instances of each relation in the table. The relations in the answer list are sorted by frequency of descending relation instance. In other words, the most frequent relation is at the top of the list.

The frequency field is a hyperlink and if followed, a new browser window shows the relation instances and a list of sentences from which each relation was extracted. In the sentences, whenever possible, the subject, relation and object are highlighted in different colors to make it easier to identify the relation and its context. Figure 3 shows the list of highlighted sentences for the semantic relation “Donepezil-TREATS-Alzheimer’s disease”. The highlighted sentences are listed in ascending order of argument-predicate distance, which is measured as the number of noun phrases between the arguments (subject and object) and the word indicating the semantic relation (the predicate). In this regard, research by Masseroli et al. [32] has shown that relations with lower argument-predicate distance have a higher likelihood of being correct. Therefore, we first show the user the relation instances that are more likely to be correct.

It is important to notice that the highlighted terms are not always the same as the official names used for the subject, relation or object. For example, in some sentences the abbreviation AD appears, but SemRep correctly recognizes this as Alzheimer’s disease. Also, the words “in” and “for” are used several times in the text to indicate treatment, which is quite frequent in medical text. This is even more common when gene symbols are mentioned in the text. Many genes have more than one symbol to denote them. And often, different genes might have the same symbol. To make things even more difficult, some gene symbols can also have another, often more common, meaning. For example, CT and MR are gene symbols, but more often mean Computed Tomography and Magnetic Resonance, respectively. This problem is known as gene symbol ambiguity and SemRep attempts to address it as described in the Background section. At the end of each highlighted sentence, the PMID of the MEDLINE citation in which the sentence appears is shown as a link that, when followed, opens the MEDLINE citation so that the context of the sentence can be seen.

We would like to stress that this view (Figure 3) also allows the user to evaluate the accuracy of the relation extraction. The last column, “Correct?”, allows the user to select whether the extraction is correct or not. If the relation has not been evaluated, the value of this field is “-“. In order to evaluate, the user has to sign in with a username and password that we provide.

Facets, if exploited, can also be considered as part of the answers. Facets convey an aggregated view of the set of answers. The type of information facets contain and their usage were described in the previous subsection and shown in Figure 2.

## Results

In this section we first describe the size of the processing involved. Then aggregated counts for the most important semantic relations and semantic types are presented, and finally, the results of the extraction correctness evaluation are shown.

### Size of processing

In the preprocessing phase we extracted semantic relations with SemRep from 122,421,765 sentences. These sentences come from 21,014,382 MEDLINE citations (the whole MEDLINE database up to the end of 2012). 13,099,644 semantic relations were extracted having a total of 58,879,300 semantic relation instances.

Table 1 shows the number of extracted relations grouped by relation name. For each name, the total number of unique relations is shown as well as the total number of instances. The relations are ordered by descending order of the number of instances. Only the top 15 semantic relations with highest instances count are shown for space saving reasons [for full table please see Additional file 1]. Knowing the semantic relation names is very important since these are the relations for which our tool is able to provide answers. The number of extracted relations and instances provide insight into which areas are better covered.

In Table 2 we show a break-down of the arguments (subject or object) of the extracted relations by semantic type. The first column shows the semantic type abbreviations which are used when formulating questions. The second column is the full name of the semantic type. The third column is the number of semantic relations in which the semantic type is the type of the argument and the fourth column is the number of instances. The semantic types are ordered in descending order by the number of instances. For space saving reasons, only the 25 most frequent semantic types are shown out of 133 semantic types that appear as arguments to relations [for full table please see Additional file 2].

### Evaluation

The quality of the answers provided in our approach largely depends on the quality of the semantic relation extraction process. Our questions must be in the form Subject-Relation-Object, and thus evaluating matching semantic relation extraction is a good (although not perfect) indicator of question-answering performance. We currently deal with a subset of all possible questions, as illustrated by the example, “Find all the drugs that inhibit the up-regulated genes from a particular microarray.” For this type of question, evaluating information extraction is very close to evaluating question answering.

Since the evaluation results shown in this paper were done for questions of the type noted above, we conducted an evaluation to estimate the correctness of the information extraction. Technically, the evaluation was done using the same QA tool used for browsing the answers, and the evaluation outcome was immediately stored in the database. The evaluation was conducted at a semantic relation instance level. In other words, the goal was to determine whether a particular semantic relation was correctly extracted from a particular sentence. The evaluators could select as outcome “correct”, “not correct” or “undecided”. Eighty subjects, students in the final year of medical school, conducted the evaluation. They were divided into four groups of twenty persons each. Each group spent three hours on an evaluation session. The subjects were organized in such a way that three of them independently evaluated the same semantic relation instance. They were not allowed to consult with each other about the outcome, and this was strictly enforced by their instructor. The idea was that each semantic relation instance included in the evaluation was to be assessed by three subjects so that voting could determine disagreement in the outcome. But in reality, since the subjects had some freedom whether to skip a relation to be evaluated and which one to evaluate from the set of assigned relations, it turned out that some instances were really evaluated by three subjects, but some were evaluated by two and some by only one person. The subjects were also instructed that the quality of the evaluation was more important than the quantity. This is probably another reason that some subjects evaluated more and some fewer relations.

In total 7,510 semantic relation instances belonging to 2,675 distinct relations were evaluated 12,083 times. The instances were evaluated as correct 8,228 times (68%) and as wrong 3,855 times (32%). 5,519 distinct instances were evaluated as correct (73%) at least once and 2,818 distinct instances were evaluated as wrong (37%) at least once. 4,692 distinct instances were always evaluated as correct (62%) and 1,991 distinct instances were always evaluated as wrong (26%). If we did not take into consideration the number of persons who evaluated a particular relation instance, we found that 4,905 (65%) distinct instances were evaluated more frequently as correct than as wrong: 2,157 (29%) instances were evaluated more often as wrong than as correct, and 448 (6%) relation instances were evaluated as correct exactly as many times as they were evaluated as wrong. If we consider only the relation instances that have been evaluated by two or more evaluators (*N* = 3,089), we found that 1,866 (60%) instances were evaluated more times as correct than as wrong, and 775 (25%) instances were evaluated more times as wrong than as correct, which means that the remaining 15% were evaluated as many times as correct as wrong. However, if we consider only the relation instances being evaluated by exactly three evaluators (*N* = 1,146), then 781 (68%) relation instances were evaluated more times as correct than as wrong, and 365 (32%) instances were evaluated more times as wrong than as correct. The relations with most instances evaluated by exactly three evaluators were INHIBITS (*N* = 865), STIMULATES (*N* = 219) and ASSOCIATED_WITH (*N* = 46) with respective extraction precisions 71%, 54% and 85%.

We calculated inter-evaluator agreement as a simple ratio between the cases where all the evaluators gave the same outcome (all correct or all wrong) divided by the total number of cases evaluated by the same evaluators. The inter-evaluator agreement for instances evaluated by two evaluators was 80% and for instances evaluated by three evaluators was 76%.

### Error analysis

We analyzed 100 system errors selected at random from predications deemed as false positives by three judges. From this analysis it became clear that incorrect answers returned by the system were largely due to erroneous predications identified by SemRep in the source documents (MEDLINE citations). Efforts are being made to address the SemRep errors noted in this research.

Slightly fewer than a quarter of the errors (21) were wrong because of concept misidentification. One subtype of this class involves failure to analyze a larger structure containing a misidentified component concept. For example, the larger structure *cyclin-dependent CDK system* was missed, and *CDK* alone was consequently interpreted as an argument of a false positive predication.

Another type of error involving concept identification is due to missing information in the Metathesaurus, specifically not having the semantic type ‘Physiologic Function’, which is one of the semantic types allowed for objects of predications with predicate STIMULATES or INHIBITS. For example, in the following sentence, the correct object of *inhibits* is *colonic aberrant crypt foci formation*, but this concept does not have semantic type ‘Physiologic Function’ and was not allowed to be the object of this predication.

*Beta-escin inhibits colonic aberrant crypt foci formation in rats and regulates the cell cycle growth by inducing p21(waf1/cip1) in colon cancer cells*.

SemRep then wrongly moved to the next concept with a semantic type allowable as an object of INHIBITS (“CDKA1A” is the Metathesaurus concept for “p21”, with semantic type ‘Amino Acid, Peptide, or Protein’) and produced the false positive “beta-escin INHIBITS CDK1A”

Four errors were due to misinterpretation of a predicate. For example, SemRep extracted the predication “Meclofenamic Acid STIMULATES ATPase” from the following sentence based on the highlighted items.

*At low glibenclamide concentrations,****MFA induced****additional inhibition of the K(****ATP****) current*.

*MFA* and *ATP* were correctly mapped to the concepts “Meclofenamic Acid” and “ATPase,” respectively. However, the correct relation between these concepts in this sentence is INHIBITS, not STIMULATES. The complex predicate *induced* … *inhibition* was not interpreted correctly (as INHIBITS), but rather *induced* was interpreted as STIMULATES by SemRep.

The rest of the errors (75) were due to various deficits in syntactic processing. We note a few of the more prominent error types in this class. Incorrect interpretation of clausal boundaries accounted for fifteen errors. Semantic predications are not allowed to cross clausal boundaries, so correct identification of this phenomenon is crucial. Intrasentential clause boundaries are commonly marked by *and* and the so-called subordinating conjunctions, such as *while* and *when*. All of these are ambiguous in that they can conjoin structures other than clauses. *And* is notorious for signaling a variety of coordination types, including, noun phrases and verb phrases, in addition to clauses. When SemRep fails to mark a clause boundary, a false positive may result. For example, SemRep extracted the predication “CDK2 INHIBITS CDKA1A” from the sentence below.

[*The levels of CDK2, CDC2, Cyclin A and Cyclin B proteins decreased*,] [*while the levels of CDK inhibitors viz., p21 and p27 were found to increase on staurosporine treatment*.]

The correct clausal boundaries for this sentence are indicated by brackets, which SemRep failed to impose. This failure then wrongly allowed *p21* (mapped to “CDKA1A”) in the second clause to be an object of “decreased” in the first clause.

Misinterpretation of passive voice, especially when truncated, accounted for 9 errors. For example, SemRep extracted the false positive predication “CDK2 INHIBITS CDKA1A” from the following sentence.

*The TB-induced cell-cycle arrest in HUVEC occurred when the cyclin-dependent kinase 2 (CDK2) activity****was inhibited****just as the protein level of p21 was increased and cyclin A was decreased*.

Correct interpretation of the passive *was interpreted* requires *CDK2* to be the object (not the subject) of *inhibited*. Further the semantic subject of *inhibited* is left unexpressed (truncated passive) in this sentence. SemRep currently does not have a facility for dealing with predications missing an argument, which is the consequence of truncated passive. The interpretation of this sentence is further complicated by the fact that SemRep failed to note the clausal boundary *just as*.

In a residue of sentences (6) with very complicated syntax, the etiology of error was due to multiple causes. For example, in the following sentence, SemRep wrongly identified the predication “Mitogen-Activated Protein Kinases INHIBITS BAG3.”

*Primary cultured astrocytes exposed to 5 mm NH4Cl for different time periods (1-72 h) significantly increased phosphorylation of extracellular signal-regulated kinase 1/2 (ERK1/2), p38(****MAPK****), and c-Jun N-terminal kinase (JNK) 1/2/3, which was****inhibited****by appropriate MAPK inhibitors 1, 4-diamino-2, 3-dicyano-1, 4-****bis****(2-aminophenylthio) butadiene (UO126; for ERK1/2), trans-1-(4-hydroxyclyclohexyl)-4-(4-fluorophenyl)-5-(2-methoxypyrimidin-4- yl)imidazole (SB 239063; for p38(MAPK)), and anthra[1,9-cd]pyrazol-6(2H)-one (SP600125; for JNK1/2/3), as well as by antioxidants*.

This predication was based on the three highlighted components: *MAPK*, *inhibited*, and *bis. MAPK* was correctly mapped to the Metathesaurus concept “Mitogen-Activated Protein Kinases,” and the verb *inhibited* is the correct indicator for the semantic relation INHIBITS. However, the text *bis* is a part of the complex chemical name in which it appears and should not have been mapped to the concept “BAG3.” Further, with respect to syntax, *MAPK* is not allowed to be an argument of *inhibited* in this sentence. In addition this verb is in passive voice (immediately preceded by *was*). Any argument to its left must be its (semantic) object, rather than subject as in the false positive being discussed.

## Discussion

Our approach to biomedical QA is, first, to extract semantic relations from MEDLINE with SemRep and build a database of these relations during a preprocessing step. Then, QA is implemented as a search in the semantic relation database. Due to the preprocessing and the appropriate database design, our approach is very fast. A typical question is answered within a few seconds, and if faceting is used or the microarray filter is switched on, the processing time takes a few additional seconds. One of the limitations of our approach is that it is currently not possible to provide contextual information when specifying the questions. For example, it is not possible to ask “What is used to treat patients with a certain disease who also have another disease or situation at the same time?” We can answer the first part of the question separately: “What is used to treat patients with a certain disease”, and the second part of the question also, but not the full question at once. One of the first items in our further work list is to allow more complex questions, which will involve combining several semantic relations in order to answer them.

We also plan to allow asking questions in natural language format, which will be useful for processing more complex questions like the one mentioned above. For less complex questions, the current approach is quite satisfactory, provided that the user spends some time learning how to formulate a question. For example, the natural language question “What drugs can be used to treat diabetes?” can be asked in our tool as “phsu treats diabetes” where “phsu” stands for “pharmacological substance” and “treats” is the name of the semantic relation. Because a lot of issues are involved with processing natural language queries, we also plan to explore another direction. We plan to develop a sophisticated user interface, based on question templates, which will allow formulating complex questions in a more structured way – a way that will be easier to process with a computer.

Since important information is present in the full text of the articles, we also plan to process a considerable number of electronically available full text articles. And since in addition to the biomedical articles there are also important websites with useful information, we also plan to process the most important such sites.

We were aware before, and the evaluation conducted for this paper has confirmed, that the semantic relation extraction process is not perfect. Therefore, improving the extraction performance is a permanent ongoing process. We believe that a very useful feature of our tool is the ability to evaluate the answers within the tool itself. In other words, whenever the user sees an answer (semantic relation) that she thinks is not correct, she can record her opinion and it is stored in our database. Right now, only the users we know and trust can evaluate, but we would like to allow any user to register and evaluate the relations. The major goal of collecting evaluation-related information is that it allows us to find the weak points and improve them. There are a few more things that can be done once we have enough evaluation data. For instance, the answers can be sorted by how many positive evaluations they have received, or they can be automatically filtered out if the users believe they are not correct. The value of this kind of evaluation is that by helping us, the users of our tool will also help themselves. This is similar to a crowdsourcing approach in which we would like to involve the biomedical community.

In the future, we also plan to conduct an evaluation of the user interface of SemBT to see how easy or difficult it is to use, and whether it is intuitive or not. Based on the results of that evaluation, we will improve the user interface accordingly.

## Conclusions

We propose a methodology and describe a tool, SemBT, for biomedical QA. The system is able to provide answers to a wide array of questions, from clinical medicine through pharmacogenomics to microarray results interpretation. SemBT is based on semantic relations extracted from the biomedical literature and is able to quickly provide precise answers to user questions. More details are provided only on demand. SemBT is publicly available at http://sembt.mf.uni-lj.si and is a useful complement to existing information retrieval systems.

## Additional files

Additional file 1:
**Semantic relations extracted with SemRep.**
Additional file 2:
**UMLS semantic types and their corresponding abbreviations that can be used for posing questions.**
